# Supplementary material for: Total small vessel disease burden and functional outcome in patients with ischemic stroke
Source: PLoS One. 2020 Nov 12;15(11):e0242319. doi: 10.1371/journal.pone.0242319 (PMC7660472; doi:10.1371/journal.pone.0242319)
Supplement: Attachment — Submitted filename: Responses to Reviewers Comments.docx [file pone.0242319.s001.docx]

| **PONE-D-20-28449 Total Small Vessel Disease Burden and Functional Outcome in Patients with Ischemic Stroke** |  |
| --- | --- |
|  |  |

**2020-10-10**

**Dear Professor Quan Jiang and Editors:**

**We thank you and referees of 'PLOS One' for taking time to review our manuscript and giving us a chance to revise the paper. We have performed additional analyses, made corrections and clarifications, highlighted in yellow, in the manuscript after going over the referees' comments. We were deeply impressed with the very constructive review, which led us to improve the quality of the manuscript substantially.**

**Reviewer #1:**

***Comment 1***

*Could please the authors explain the reason for not adjusting the multivariate model with the NIHSS score? Has this analysis been performed? It is a common predictor of functional outcome in patients with stroke and patients with SVD 4 tend to have higher NIHSS scores (p75 is 10).*

**Reply 1**

We thank the reviewer for this critical comment. As the reviewer pointed out, we additionally included NIHSS scores in the revised multivariable model. After further adjusting for NIHSS scores, the association between total SVD scores and 3-month mRS scores materially unchanged. In addition, the association between each SVD marker and 3-month mRS scores remained significant.

We amended the statistical analysis section in method, and Table 2 follows.

Method: Pre-defined covariates potentially associated with functional outcome after ischemic stroke, including age, sex, pre-stroke mRS score, initial NIHSS scores, previous history of stroke, hypertension, diabetes, smoking, coronary artery disease, stroke subtypes, and revascularization, were entered into the multivariable model.

**Table 2. Impact of total small vessel disease score and 3-month modified Rankin Scale score**

|  | Adjusted odds ratio (95% confidence interval) | P value |
| --- | --- | --- |
| Total SVD score |  |  |
| 0 | Reference |  |
| 1 | 0.96 (0.58 – 1.58) | 0.87 |
| 2 | 1.73 (1.05 – 2.83) | 0.031 |
| 3 | 2.18 (1.22 – 3.88) | 0.008 |
| 4 | 2.21 (1.04 – 4.69) | 0.038 |
| P for trend | 0.008 | |
| Presence of lacune | 1.63 (1.13 – 2.38) | 0.009 |
| Presence of CMBs | 1.88 (1.28 – 2.76) | 0.001 |
| Presence of EPVS | 1.19 (0.84 – 1.70) | 0.32 |
| Above median of WMH volume* | 1.87 (1.25 – 2.81) | 0.002 |

Adjusted for age, sex, initial National Institute Health stroke scale scores, pre-stroke mRS score, previous history of stroke, hypertension, diabetes, smoking, coronary artery disease, stroke subtypes, and revascularization.

Multivariable ordinal logistic regression analysis was performed for each dependent variable.

* Median = 0.85% of total brain volume

SVD indicates small vessel disease; CMB, cerebral microbleed; EPVS, enlarged perivascular space; WMH, white matter hyperintensity.

***Comment 2***

*Some of the English writing should be reviewed.*

**Reply 2**

A native English speaker edited the revised manuscript. Regarding this, we added acknowledgment.

Acknowledgments

We thank Dr. Jung E Park for assistance with English language editing.

***Comment 3***

*The authors use many times in the manuscript the expression "different subtypes of SVD" when they actually refer to different neuroimaging markers of SVD. I suggest use the expressions "SVD markers" or "neurimaging markers of SVD" instead. The "use of subtypes of SVD" can cause confusion to the readers, since SVD has several underlying etiologies such as hypertensive, amyloid angiopathy, CADASIL.. and the expression "SVD subtypes" is frequently used in this setting.*

**Reply 3**

We appreciate this valuable comment. We agree with the reviewer's opinion. In the revised manuscript, the expression "subtypes of SVD" were replaced by "SVD markers" or "neuroimaging markers of SVD."

***Comment 4***

*The authors perform a multivariate ordinal regression analysis. Therefore, it would be more correct if they say that "lacunes, MBS… independently increased the risk of worsening on the mRS scale" instead of "lacunes, MBS….were independently associated with unfavorable outcome", because they analyze the whole spectrum of the scale and not only the unfavorable outcome categories(3-6).*

**Reply 4**

As the reviewer pointed out, we revised the phrase throughout the entire manuscript to clearly describe the results.

For example, the sentence "However, EPVS was not associated with 3-month outcome" was replaced with "However, EPVS was not associated with 3-month mRS scores."

***Comment 5***

*Why have the authors selected the period 2012 to 2013 for the analysis of 90 days mRS?. Don't they have more recent data?*

**Reply 5**

The present study cohort is derived from the Korean Brain MR database comprised of about 13,000 ischemic stroke patients who were admitted to 11 stroke centers in Korea from 2011 to 2013. Although our center is continuing to collect brain MRI of stroke patients, a stroke neurologist's image review is currently completed in patients admitted until 2013.

***Comment 6***

*How many centers participate in the study?. It is not clear from the methods section. At the beginning of this section, the authors say" patients who visited our center", but lately they say "stroke subtypes were determined by the consensus experienced neurologists in each participating center".*

**Reply 6**

The present study is based on single-center data. We regret that our careless statement leads the reviewer to misunderstand. In the revised manuscript, we clarified this issue by adding the statement below in the method and deleting the phrase "in each participating center."

We performed post hoc analysis on prospectively-collected data from a single-center stroke registry.

**Reviewer #2:**

***Comment 1***

*Please report in the abstract number and strength of association rather than p values*

**Reply 1**

Thank you for the comment. As the reviewer pointed out, we revised the results section in the abstract as follows.

The proportion of mRS scores 2 or greater was 16% and 47% in total SVD score 0 and 4, respectively. Multivariable ordinal logistic regression analysis results showed that compared with the total SVD score of 0, total SVD scores of 2, 3, and 4 were independently associated with higher mRS scores with adjusted odds ratios (95% confidence intervals) of 1.68 (1.02-2.76), 2.24 (1.25-4.00), and 2.00 (1.02-4.29). Lacunes, CMBs, WMHs but not EPVS were associated with mRS scores at 3 months. However, the impact of each SVD marker on stroke outcome was smaller than that of the total SVD score.

***Comment 2***

*Please present general characteristics of population in the first part of results (age, sex, median NIHSS, etc.)*

**Reply 2**

Following the reviewer's comment, we inserted a description of the population's general characteristics in the first paragraph of the results as follows.

Among 477 patients included, the mean age was 66.3 (standard deviation 14.1), 38% were women (n = 183), the median NIHSS 3 (interquartile range 1–5), and the median WMH volume was 0.85% of total brain volume (interquartile range 0.39–1.80).

***Comment 3***

*Please state inclusion and exclusion criteria for study population. E.g. were included patients treated with acute stroke treatment?*

**Reply 3**

We appreciate the valuable comment. We included consecutive patients who visited our stroke center within 7 days of symptom onset and were confirmed as ischemic stroke based on brain MRI. With regard to this, we revised the second sentence of the method as below.

We performed post hoc analysis on prospectively-collected data from a single-center stroke registry. From February 2012 to September 2013, we included a total of 687 patients who visited our center within 7 days of stroke onset and were confirmed to have had ischemic stroke based on brain MRI.

***Comment 4***

*Please move the excluded patients paragraph from methods to results section*

**Reply 4**

Following the reviewer's comment, we moved the paragraph to the first paragraph of the results section.

Among 687 patients who were screened, we excluded those with the following to calculate the total SVD score; patients with incomplete MRI sequences(n = 135), poor MRI quality (n = 32), and lost to follow-up (n = 43), leaving 477 patients for the analyses.

***Comment 5***

*a single reader rated all the scans*

**Reply 5**

We appreciate the comment. In order to maintain a consistent rating, all images have been reviewed and rated by one reader who has experienced in a neuroimaging study. Indeed, the guideline (*Neuroimaging standards for research into small vessel disease and its contribution to ageing and neurodegeneration. Wardlaw et al. Lancet Neurol. 2013*) recommended that "For all lesion types, observers should be trained in neuroimaging interpretation, and specifically how to recognise lesions of SVD. If possible, particularly in trials or large observational studies, images should be rated centrally by one or a few experienced raters. In studies with more than one rater, inter-rater reliability should be assessed". Therefore, we assumed that a single reader is not a limitation of our study.

***Comment 6***

*Table 1 needs some editing (e.g. age, years, probably "mean" is missing). Please add a column with data from all the population.*

***Reply 6***

Thank you for pointing this out. To clearly distinguish the data presentation, we changed the data presentation from mean (standard deviation) to mean ± standard deviation. We also amended the footnote of Table 1. Following the suggestion, we added a new column with data from all patients.

***Comment 7***

*What "revascularization" means in the adjusted analysis?*

**Reply 7**

We did include patients who underwent recanalization therapy, either intravenous tPA or mechanical thrombectomy, or both. Our center uses CT perfusion and CT angiography for the patients who are eligible for thrombectomy. In severe strokes or the failed revascularization, the patients may not be possible to take brain MRI due to the unstable medical condition, so they are excluded from the study.

Together with comment 11 by the second reviewer, we amended the limitation section. Please refer to reply 11.

***Comment 8***

*Although stroke severity (i.e. NIHSS) is a major determinant of stroke outcome, NIHSS is missing in the adjusted analysis. In table 1 there is a clear trend towards worse NIHSS in patients with higher SVD burden. Conversely, in the adjusted analysis there are some variables that likely do not affect stroke outcome (e.g. smoke exposure, coronary heart disease-anamnestic, I suppose-). I would like to see a further multivariable analysis adjusted simply for age, sex, NIHSS, pre-stroke mRS.*

**Reply 8**

Another reviewer has raised the same issue. We agree with the reviewers' opinion. Thus, in the revised manuscript, we included NIHSS as an additional covariate. Additionally, as the reviewer pointed out, we performed a further multivariable analysis in which only age, sex, NIHSS, pre-stroke mRS were included as covariates. Please see the table below. The association between total small vessel disease scores and 3-month mRS scores did not change materially in the new model.

Table. Simple multivariable analysis between total small vessel disease score and 3-month modified Rankin Scale score

|  | Adjusted odds ratio (95% confidence interval) | P value |
| --- | --- | --- |
| Total SVD score |  |  |
| 0 | Reference |  |
| 1 | 1.07 (0.65 – 1.74) | 0.80 |
| 2 | 1.71 (1.05 – 2.80) | 0.032 |
| 3 | 2.25 (1.29 – 3.92) | 0.004 |
| 4 | 2.22 (1.06 – 4.68) | 0.035 |
| P for trend | 0.012 | |

Adjusted for age, sex, admission National Institute Health stroke scale scores, and pre-stroke mRS score. SVD indicates small vessel disease;

***Comment 9***

*Figure 1 is rather confusing*

**Reply 9**

Thank you for the comment. We added more explanation for the figure in the legend as below.

The prevalence of other SVD markers in each SVD marker. 'Yes' and 'No' indicate the presence and absence of each SVD marker, respectively. In all SVD markers, other makers were more prevalent in patients with each SVD marker. All P values < 0.05 (chi-square test). CMB indicates cerebral microbleed; WMH, white matter hyperintensity; EPVS, enlarged perivascular space; SVD, small vessel disease

***Comment 10***

*Discussion: there are also reports about no association between SVD and functional outcome after stroke (e.g. Boulouis G et al., Neurology 2019), please discuss your results comparing also with such studies.*

**Reply 10**

We appreciate the reviewer's suggestion. We added more discussion about this issue as follows.

In contrast, a study from patients receiving endovascular treatment demonstrated that WMHs were not associated with outcomes after stroke [22]. Different study populations, different sample sizes may be possible reasons for this discrepancy.

***Comment 11***

*Discussion: as a limit, authors mentioned that patients that were eligible for endovascular treatment were excluded from the study. However, there are reports about SVD and outcomes in such patients (Arba F et al. Neurol Sci 2019; Boulouis G et al., Neurology 2019). Again, what about patients eligible for intravenous rt-PA (see Charidimou A et al., Stroke 2016; IST-3 collaborators, Lancet Neurol; Arba F et al., Acta Neurol Scand 2017)?*

**Reply 11**

Thank you for this valuable comment. Mechanical thrombectomy was not an exclusion criterion in our study. Our center uses CT perfusion and CT angiography for the patients who are eligible for thrombectomy. Furthermore, patients with thrombectomy were likely to experience severe stroke to be excluded from the study.

To clarify and discuss this issue, we amended the paragraph regarding limitations as follows. We added references the reviewer recommended.

First, patients with severe stroke and revascularization therapy may have been excluded from the study. As a result, this selection bias may underestimate the degree of association between total SVD burden and function outcomes because SVD is associated with functional outcomes in patients who received thrombectomy [Arba F, et al. Neurol Sci. 2019] and intravenous recombinant tissue plasminogen activator [Arba F et al. Acta Neurol Scand. 2017].

***Comment 12***

*Results apply only to patients who underwent MR, but the great majority of stroke patients receive only CT. Please discuss.*

**Reply 12**

We agree with the reviewer's opinion. We inserted the statement below as one of the limitations.

Third, because the present study included only patients with MRI, our results have limited generalizability to patients who undergo only brain computed tomography (CT).
